# LNMSNet: a multi-task deep learning network for pulmonary nodules segmentation and malignancy classification

**DOI:** 10.3389/fmed.2026.1773338

**Published:** 2026-03-30

**Authors:** Yuxin Liu, Zhenyu Tang, Zhenkun Tang, Junlai Qiu, Rong Zheng, Zhong Tang, Yuexiang Li

**Affiliations:** 1 School of Information and Management, Guangxi Medical University, Nanning, Guangxi, China; 2 Department of Electrical Engineering and Computer Science, University of California, Irvine, Irvine, CA, United States; 3 Information Department, Guangxi Medical University Affiliated Tumor Hospital, Nanning, Guangxi, China; 4 Medical AI ReSearch (MARS) Group, Center for Genomic and Personalized Medicine, Guangxi Key Laboratory for Genomic and Personalized Medicine, Nanning, Guangxi Collaborative Innovation Center for Genomic and Personalized Medicine, University Engineering Research Center of Digital Medicine and Healthcare, Guangxi Medical University, Nanning, Guangxi, China; 5 School of Humanities and Social Sciences, Guangxi Medical University, Nanning, Guangxi, China

**Keywords:** classification, lung cancer, multi-scale feature extraction, multi-task learning, segmentation

## Abstract

**Introduction:**

Lung cancer remains the leading cause of global cancer incidence and mortality, with late-stage diagnosis contributing to a stark 5-year survival rate of only 8%. Systematic early detection via low-dose computerized tomography (LDCT) scan can dramatically improve outcomes, with survival exceeding 90% for stage I patients. Pulmonary nodules are the primary radiological precursor, but their accurate characterization is challenged by manual interpretation, inter-reader variability, and the difficulty of visually assessing small, ill-defined lesions on hundreds of CT slices.

**Methods:**

To this end, we propose LNMSNet, which extracts Multi-Scale features from Lung Nodules for the joint segmentation and malignancy classification. The model employs a U-shaped encoder-decoder with a ResNet-18 backbone. A key innovation of our LNMSNet is the MSConv module, which uses parallel multi-scale convolutions to capture both fine-grained texture and global contextual features, thereby enlarging the receptive field and improving boundary accuracy and size invariance.

**Results:**

We validated the proposed LNMSNet on a multi-center external dataset of 220 CT scans from two tertiary hospitals. The model showed superior performances in both tasks compared to other multi-task models and exhibited stable generalizability across institutions.

**Conclusion:**

The proposed LNMSNet effectively leverages multi-scale feature extraction and joint task optimization for accurate pulmonary nodule characterization.

## 1 Introduction

GLOBOCAN 2022 reports that lung cancer accounts for 12.4% of all new cancers and 18.7% of cancer deaths worldwide, ranking first in both incidence and mortality (1). The 5-year survival rate for advanced disease is 8%; 70%–90% of these patients die within 5 years (2). Systematic early detection reverses this outlook, yielding a 5-year survival of 90.4% for stage I patients (3). Lung/pulmonary nodules are the principal imaging precursor; therefore accurate classification of a nodule as benign or malignant is the critical step in lung-cancer screening. Early-stage tumors are clinically silent, so low-dose CT is the standard detection tool. Yet interpretation is still manual: a single study contains over 100 slices, and nodules are often smaller than 10 mm, ill-defined, or adjacent to vessels and inflammatory infiltrates, making visual feature extraction difficult (4, 5). In addition, radiologists differ in their assessment of density, spiculation, and calcification, producing inter-reader variability that can alter management (6).

Deep learning has been comprehensively integrated into computer-aided diagnosis (CAD) for pulmonary nodules analysis, demonstrating proficiency across multiple tasks. For instance, Ardila et al. (7) proposed an algorithm that utilizes both current and prior computed tomography volumes to predict lung cancer risk, achieving radiologist-level sensitivity for nodule detection. Furthermore, Liu et al. (8) processed 12,754 thin-section CTs and obtained higher sensitivity than unassisted readers while halving the reading time. For medical image segmentation, Isensee et al. (9) showed that a systematically configured U-Net could match or surpass specialized networks, highlighting that gains from optimized training protocols often exceed those from architectural modifications. For classification, Ciompi et al. (10) demonstrated that deep learning systems could effectively discriminate benign from malignant nodules on routine CT scans, offering validated diagnostic support. Collectively, these studies confirm that deep learning can localize, segment, and characterize nodules; however, each system is typically optimized for a single task, revealing a key limitation in the current paradigm.

To overcome these limitations, multi-task learning (MTL) frameworks share features and jointly train detection, segmentation, and classification, cutting parameter redundancy and information isolation of single-task models (11). Ni et al. (12) cascade coarse-to-fine segmentation with classification to refine nodule contours. Qi et al. (13) feed one CT slice into a convolutional neural network (CNN) that simultaneously segments the lesion and predicts the histological subtype. Tang and Zhang (14) share encoder weights between segmentation and classification and outperform baselines of a single-task. However, the evaluation is largely confined to the public LIDC-IDRI (15) dataset; evidence from routine clinical CT is scarce (16). In medical image analysis, lesion regions often exhibit complex characteristics due to their variability in size, shape, and texture. Traditional convolutional methods, limited by their receptive field, struggle to capture both fine details and macro structural information simultaneously (17). This limitation poses challenges for models in accurately delineating and capturing the intricate boundaries and morphology of lesions.

We propose a 3D single-stage multi-task network (termed LNMSNet), which can extract Multi-Scale features from Lung Nodules, to jointly segment pulmonary nodules and classify malignancy. The model uses a U-shaped encoder-decoder (18) with ResNet-18 (19) as the shared encoder. To capture texture details and global structure simultaneously, we design MSConv, a parallel multi-scale convolution module that enlarges the receptive field and fuses multi-scale features, improving boundary accuracy and size invariance. We collect 220 cases multi-center CT scans from two tertiary hospitals for external validation. LNMSNet on the external set, outperforming other multi-task models and demonstrating stable performance across centers. In summary, our contributions are: (1) an end-to-end 3D multi-task network is proposed for nodule segmentation and malignancy classification; (2) a novel MSConv module is designed to enhance multi-scale feature fusion; (3) an external validation is conducted on 220 cases clinical scans confirming the generalizability of our LNMSNet.

## 2 Related works

### 2.1 Segmentation of pulmonary nodules in CT images

Early methods use thresholding (20), region growing (21), watershed (22), or edge detection. They rely on gray-level differences without 3-D context, so segmentation degrades noticeably in the presence of noise, vessel attachment or blurred margins, and each new dataset requires manual retuning of multiple parameters. Mukherjee et al. (23) pioneered early hybrids that married deep-learned priors with conventional algorithms. Wang et al. (24) propose a central-focus CNN that mimics radiologists fixation on the nodule core. Jiang et al. (25) introduce a multi-resolution residual stream to fuse global and local cues locate tumor regions. Chen et al. (26) propose a multi-crop convolutional network that processes overlapping local patches in parallel and aggregates multi-scale context to achieve high segmentation accuracy. The fully convolutional 3D nested network developed by Kido et al. (27) integrates residual unit structures and is capable of capturing complex spatial features. Addressing the issues of data scarcity and class imbalance in medical imaging. Tyagi and Talbar (28) employ a 3D GAN for data augmentation to improve model generalization under small-sample conditions. Recent innovations have tackled distinct aspects of segmentation. For boundary integrity, Wang et al. (29) combined a hybrid Transformer with boundary enhancement, while Cai et al. (30) developed a multi-level dynamic fusion network. Bhattacharjee et al. (31) addressed efficiency and heterogeneity via a double-skip connection structure enabling 43-min training. Separately, Yang et al. (32) boosted reliability with an uncertainty-aware attention mechanism. To address nodules with significant scale variations, Yin et al. (33) proposed an atrous multi-scale convolution attention module and applied it to the downsampling encoder to expand the receptive field by paralleling convolution kernels with different dilation rates, thereby achieving segmentation of targets at different scales. Rahman and Marculescu (34), starting from multi-scale features of multi-branch convolution, proposed a lightweight module that parallels convolution kernels of different sizes to capture multi-resolution information. However, although the former has a rich receptive field which improves feature extraction efficiency, it neglects edge information due to limited detail capture; while the latter achieves multi-kernel parallelism, it suffers from insufficient contextual modeling due to large-range feature extraction. Collectively, these studies deliver specialized models for boundaries, efficiency, and robustness, yet they remain singular solutions that do not leverage the potential synergies of multi-task learning.

### 2.2 Classification of pulmonary nodules from CT images

Deep-learning models now routinely outperform conventional machine-learning pipelines in distinguishing benign from malignant pulmonary nodules. Liu et al. (35) encoded multi-view and multi-scale CT information within a single convolutional graph, improving discrimination across heterogeneous nodular morphologies. Subsequent works (36–38) have consistently shown that fusing these data-driven representations with clinician-curated semantic labels or classical radiomic signatures captures complementary signal, yielding higher and more stable AUCs than either source alone. Alongside feature fusion, architectural refinements have also boosted performance. For example, Zhang et al. (39) developed an SE-ResNeXt-based model that achieved strong results on the LUNA16 dataset by leveraging channel-wise attention and multi-branch feature learning. When labeled volumes are scarce, Shi et al. (40) demonstrated that a curriculum mixing transfer learning with semi-supervised consistency regularization leverages thousands of unannotated scans to refine decision boundaries. Huang et al. (41) pushed this idea further via self-supervised pre-training followed by domain-adversarial fine-tuning (SSTL-DA), sustaining high sensitivity even when only a handful of annotated cases are available. Architectural innovations have also proven effective: Mobiny et al. (42) replaced standard convolutional blocks with memory-augmented capsules, allowing rapid adaptation to new institutional distributions with minimal labeled data. Saied et al. (43) on the other hand, used radiomics features of pulmonary nodules to construct a deep transfer learning model for more efficient classification of benign and malignant tumors. The 3D Attention Gated Convolutional Network (AG-Net) proposed by Liu et al. (44) combines nodule features, surrounding microenvironment, and fibrotic semantic information to further enhance model performance.

### 2.3 Multi-task learning framework of medical images

Multi-task deep learning (MTDL) has been increasingly introduced into computer-aided diagnosis to exploit the synergy between tasks, thereby alleviating the limitations of single-task models in terms of accuracy and generalization. Early studies mainly focused on sharing feature representations through joint learning of multiple tasks. For example, Liu et al. (45) proposed a unified framework for lung parenchyma segmentation and pulmonary nodule detection, where a pyramid dilated convolution module was introduced to enlarge the receptive field while preserving fine-grained details and suppressing the grid artifacts inherent in standard dilated convolutions.

Building upon this, subsequent studies have paid more attention to enhancing information interaction between tasks through feature sharing mechanisms. Wang et al. (46) proposed an adversarial multimodal fusion network with cross-modal attention for skin lesion analysis, where low-level texture information is shared between segmentation and classification branches to achieve accurate diagnosis. In another work, Wang et al. (47) introduced an interpretable MTDL model based on the information bottleneck principle, which constrains feature flow to tumor-related regions, and further coordinates the two tasks through dual-prior regularization, thereby improving localization and discrimination performance. Similarly, van der Voort et al. (48) designed a single CNN to simultaneously perform glioma segmentation, molecular subtyping, and tumor grading, demonstrating that the gradient updates from one task can stabilize the feature space of other tasks. Subsequently, attention mechanisms have been incorporated into multi-task frameworks to further enhance feature representation capability. Ling et al. (49) developed a single-stage multi-task attention network (MTANet) equipped with reverse attention, where global semantics explicitly enhance local details and high-resolution boundary features, leading to improved overall understanding of the image. In addition, Lei et al. (50) proposed a hippocampus-guided multi-task model that introduces anatomical priors to guide the network to focus on disease-related regions, enabling joint disease classification, hippocampus segmentation, and clinical score regression. Zhang et al. (51) and Ma et al. (52) both adopt multi-attention guidance and multi-scale feature fusion strategies to improve multi-task learning performance. The former focuses on gastric tumor segmentation and lymph node classification, while the latter is designed for glioma segmentation and IDH genotype prediction. Zhou et al. (53) further developed an end-to-end multi-task learning framework for breast ultrasound image segmentation and classification, employing an iterative training strategy to progressively refine feature representations and highlight tumor regions for both tasks.

Despite these advances in multi-task collaboration and attention modeling, several limitations remain: (1) existing methods mainly focus on enhancing feature representation through attention mechanisms or incorporating prior knowledge, while insufficiently modeling the significant scale variations of targets (e.g., pulmonary nodules ranging from a few millimeters to several centimeters). (2) Feature sharing in multi-task frameworks may introduce conflicts between tasks. In particular, when segmentation and classification are jointly optimized, relying on single-scale or single-form attention mechanisms may cause the model to overly focus on local regions, thereby affecting the global structural representation and the stability of segmentation results. Therefore, how to simultaneously incorporate multi-scale contextual modeling and effective feature selection within a multi-task learning framework, in order to alleviate feature conflicts between tasks and improve the adaptability of the model to lesions of varying sizes, remains an important problem to be addressed.

## 3 Methodology

### 3.1 Overview of LNMSNet

**Figure 1:**
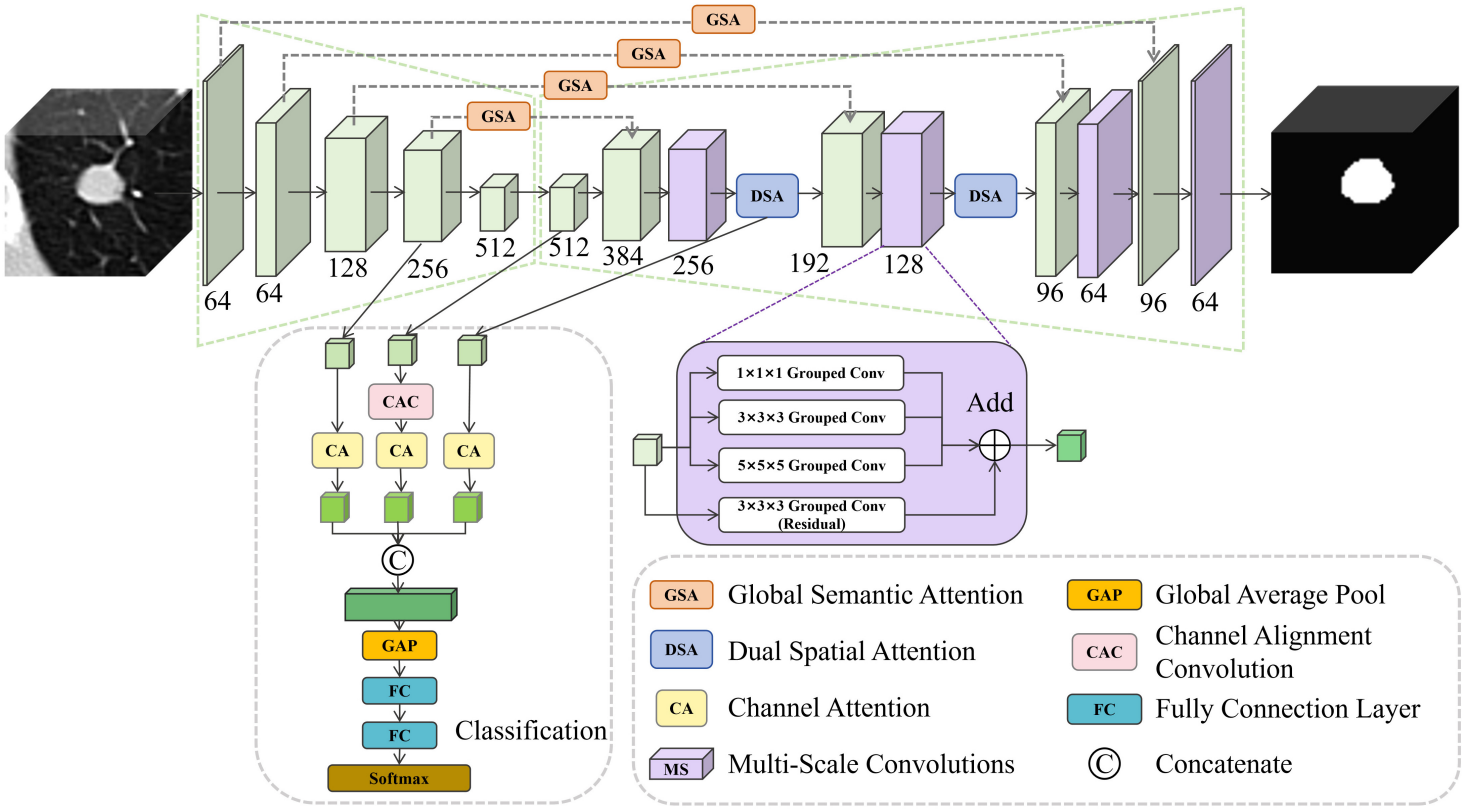
The pipeline of the proposed LNMSNet. Our LNMSNet is consisted of multiple novel modules, e.g., Global Semantic Attention and Multi-Scale Convolutions, for accuracte lung segmentation and malignancy classification.

We propose a 3D multi-scale attention-guided multi-task network, termed LNMSNet, which adopts ResNet-18 as the backbone and follows an encoder-decoder architecture. A classification branch is connected to the high-level encoder features and incorporates a channel attention (CA) module to fuse mid-to-high encoder features with early fused decoder representations for malignancy prediction. The nodule CT patch is first fed into the encoder, where four residual blocks progressively downsample the features to form hierarchical feature maps. These maps are then delivered to the decoder for segmentation and to the classification branch for benign-malignant prediction. Meanwhile, features from each encoder stage are transferred to the decoder via skip connections and fused with the corresponding decoder features. Before fusion, the transmitted features pass through the Global Semantic Attention (GSA) module, which adaptively reweights the responses and suppresses less relevant activations. Within the decoder, multi-scale convolution modules are employed to capture multi-scale contextual information, enhancing the decoder's sensitivity to nodules of different sizes and maintaining awareness of both local details and global structures while restoring spatial resolution. Finally, after high-level feature fusion in the decoder, Dual Spatial Attention (DSA) modules are introduced to generate spatial attention maps via channel-wise average and max pooling, guiding the network to focus on critical nodule regions, thereby producing richer and more robust segmentation features. The overall pipeline is depicted in Figure 1.

### 3.2 Semantic and spatial attention modules

To control the quality of information transmitted through skip connections and prevent irrelevant or conflicting responses from being directly propagated and amplified, inspired by He et al. (54), we introduce a Global Semantic Attention (GSA) module to selectively reweight skip-connection features. Specifically, the input feature map is first projected and compressed through convolutional layers followed by group normalization and ReLU activation to generate two intermediate response maps. These response maps are activated by a Sigmoid function and aggregated via global average pooling and global max pooling, respectively, producing global descriptors that summarize channel-wise responses. The obtained descriptors are then used to adaptively reweight the original features, emphasizing task-relevant information while suppressing less informative responses. Finally, the reweighted features are further refined through normalized and nonlinear convolutional transformations, resulting in more stable and discriminative representations for subsequent decoding.

To enhance the model's focus on lesion-relevant regions and improve pulmonary nodule segmentation accuracy, we incorporate a Dual Spatial Attention (DSA) module in the first two decoder stages. Unlike attention mechanisms based on a single pooling strategy, DSA adopts parallel average and max pooling branches to extract complementary spatial responses. The average pooling branch aggregates channel responses to emphasize spatial regions consistently activated across channels, which helps stabilize the localization of nodule cores. In contrast, the max pooling branch highlights locally strong activations, contributing to clearer boundary delineation. These two spatial descriptors are then processed through convolution and Sigmoid activation to generate spatial weighting maps that rescale the features adaptively. By combining consensus-based and peak-based spatial cues, the module allows the decoder to attend to both internal structures and boundary details of pulmonary nodules. However, as its attention modeling is primarily based on single-scale spatial responses, DSA does not explicitly capture multi-scale contextual information, which may limit robustness when segmenting nodules of varying sizes in complex scenarios.

### 3.3 Multi-scale convolution (MSConv) module

GSA and DSA modules optimize the feature flow from semantic and spatial dimensions, respectively. However, in real clinical scenarios where pulmonary nodules exhibit substantial scale variations—from a few millimeters to several centimeters—these mechanisms alone are insufficient to fully address such diversity. This limitation may reduce the model's sensitivity to small nodules or result in incomplete structural representation of larger lesions. To alleviate this issue, we introduce a Multi-Scale Convolution (MSConv) module and embed it after decoder feature fusion and before the DSA module. MSConv combines multi-scale feature extraction with residual connections, employing parallel multi-branch dilated convolutions to capture contextual information across different spatial scales. By extracting features under varying receptive fields, the decoder is better equipped to adapt to nodules of diverse sizes and morphologies.

Specifically, the MSConv module takes a three-dimensional feature map $X\in {\mathbb{R}}^{C_{\text{in}}\times D\times H\times W}$ as input, where *D*, *H*, and *W* denote the depth, height, and width of the feature map, and *C*_in_ is the number of input channels. The input feature map is processed in parallel by four grouped convolutional branches, including three multi-scale grouped convolutions with kernel sizes 1 × 1 × 1, 3 × 3 × 3, and 5 × 5 × 5, as well as a residual grouped convolution with a kernel size of 3 × 3 × 3. All branches share a unified group number *g*, which is adaptively determined as the largest integer satisfying *g* ≤ min(⌊*C*_in_/2⌋, *C*_out_) and *g*∣*C*_out_, thereby enabling efficient grouped convolution while preserving intra-group channel interaction. Specifically, the 1 × 1 × 1 branch focuses on lightweight feature transformation and intra-group channel interaction. The 3 × 3 × 3 branch captures local spatial structures and extracts mid-range contextual information, while the 5 × 5 × 5 branch enlarges the receptive field to incorporate broader spatial context. The residual 3 × 3 × 3 grouped convolution provides a stable information-preserving path, facilitating effective gradient propagation and efficient aggregation of multi-scale features. These branches produce feature maps *F*_1_, *F*_2_, *F*_3_, and $F_{\text{res}}\in {\mathbb{R}}^{C_{\text{out}}\times D\times H\times W}$. Subsequently, the three multi-scale feature maps *F*_1_, *F*_2_, and *F*_3_ are summed element-wise to obtain a fused representation, which is then combined with the residual feature map *F*_res_ via element-wise addition to generate the final output feature map $Y\in {\mathbb{R}}^{C_{\text{out}}\times D\times H\times W}$. The overall computation process of the MSConv module can be expressed as Equation 1:


$$\begin{array}{l} Y=\text{Con}{\text{v}}_{\text{res}}^{\left(g\right)}\left(X\right)+\underset{k\in \left\{1,3,5\right\}}{\sum }\text{Con}{\text{v}}_{k}^{\left(g\right)}\left(X\right). \end{array}$$ (1)

All convolutional branches adopt grouped convolution with a shared group number *g*, defined as the largest divisor of the output channel dimension. $\text{Con}{\text{v}}_{k}^{\left(g\right)}$: grouped convolution with kernel size *k*×*k*×*k*. $\text{Con}{\text{v}}_{\text{res}}^{\left(g\right)}$: residual grouped convolution for stable feature propagation. This multi-scale convolution design allows the MSConv module to aggregate contextual information across different receptive fields while keeping the computational overhead modest. As a result, the decoder is better equipped to handle scale variations commonly observed in pulmonary nodules, leading to more stable and reliable feature representations for detection and segmentation tasks.

### 3.4 Classification branch

In the design of the classification head, LNMSNet follows the common practice of CNN-based image classification networks that exploit high-level feature representations (19, 55). A classification branch is inserted between the encoder and decoder, where intermediate-to-high-level and high-level features from the last two residual stages of the encoder, together with early fused features from the decoder, are fed into the classification branch. To alleviate feature conflicts across different hierarchical levels, channel attention modules are introduced to adaptively select and reweight features from each input stream, thereby emphasizing features that are more informative for classification. Through multi-level feature integration and attention-guided selection, the classification branch effectively captures rich semantic information for pulmonary nodule malignancy prediction.

### 3.5 Loss function

To optimize both segmentation and classification tasks within our multi-task framework, we design a compound loss function consisting of a segmentation loss and a classification loss. The total loss is expressed as Equation 2:


$$\begin{array}{l} L_{\text{total}}=L_{\text{seg}}+L_{\text{cls}}, \end{array}$$ (2)

where $L_{\text{seg}}$ and $L_{\text{cls}}$ denote the segmentation loss and classification loss, respectively. For the segmentation branch, we adopt a hybrid loss combining Cross-Entropy (CE) loss and Dice loss as Equation 3:


$$\begin{array}{l} L_{\text{seg}}=L_{\text{CE}}+L_{\text{Dice}}. \end{array}$$ (3)

The CE loss is defined as Equation 4:


$$\begin{array}{l} L_{\text{CE}}=-\frac{1}{N}\overset{N}{\underset{i=1}{\sum }}\overset{C}{\underset{c=1}{\sum }}y_{i,c}\log p_{i,c}, \end{array}$$ (4)

where *p*_*i, c*_ denotes the softmax probability of voxel *i* belonging to class *c*, *y*_*i, c*_ is the corresponding one-hot ground truth label, *N* is the number of voxels, and *C* is the number of classes (background and nodule). Dice loss is computed on the foreground (nodule) class to explicitly constrain lesion overlap. It is formulated as Equation 5:


$$\begin{array}{l} L_{\text{Dice}}=1-\frac{2\sum_{i}p_{i,1}y_{i,1}+\epsilon }{\sum_{i}p_{i,1}+\sum_{i}y_{i,1}+\epsilon }, \end{array}$$ (5)

where *p*_*i*, 1_ is the softmax probability of voxel *i* belonging to the nodule class, *y*_*i*, 1_ is the corresponding binary foreground mask, and ϵ = 10^−5^ is a smoothing constant for numerical stability.

The classification branch is optimized using the standard cross-entropy loss (Equation 6):


$$\begin{array}{l} L_{\text{cls}}=-\frac{1}{B}\overset{B}{\underset{b=1}{\sum }}\overset{2}{\underset{c=1}{\sum }}y_{b,c}^{\text{cls}}\log p_{b,c}^{\text{cls}}, \end{array}$$ (6)

where $p_{b,c}^{\text{cls}}$ denotes the predicted probability of sample *b* belonging to class *c* (benign or malignant), $y_{b,c}^{\text{cls}}$ is the corresponding ground truth label, and *B* is the batch size. This joint optimization encourages the shared encoder to learn complementary semantic representations that benefit both voxel-level segmentation and patient-level malignancy prediction.

## 4 Experimental results

### 4.1 Dataset

This study evaluates the segmentation and classification performance of LNMSNet using three datasets: Two private contrast-enhanced CT cohorts collected from collaborating hospitals and the publicly available LIDC-IDRI database. The two private datasets consist of CT scans from 224 patients (155 from Hospital A and 69 from Hospital B), yielding 242 pulmonary nodules collected between 2022 and 2025. Surgical pathology confirmed 84 benign and 159 malignant nodules. The scans were acquired using five CT systems from different manufacturers, with specifications summarized in Table 1, covering configurations ranging from 64-slice to 320-slice scanners. All images were reconstructed as thin-slice lung window scans with slice thicknesses between 0.6 and 2.0 mm, and each CT image had a matrix size of 512 × 512 pixels. The diameters of the annotated nodules ranged from 3 to 30 mm. All lesions were manually annotated by two senior thoracic radiologists using ITK-SNAP to obtain voxel-wise ground-truth masks. The overlapping regions of the two annotations were used as the reference standard for segmentation, while surgical pathology served as the ground truth for determining nodule malignancy.

**Table 1:** CT acquisition and reconstruction parameters used in the private external validation cohorts from two hospitals.

Parameters		Hospital A			Hospital B	
CT	CT system	128 slice spiral	64 slice spiral	320 slice spiral	128 slice spiral	64 slice spiral
	Manufacturer	GE	SIEMENS	United imaging	GE	TOSHIBA
	Tube voltage	120 kVp	120 kVp	120 kVp	100 kVp	120 kVp
	Tube current	4 mAs	180 mAs	170 mAs	5 mAs	89 mAs
	Rotating time	0.7 s	0.5 s	0.5 s	0.7 s	0.5 s
	Detector	64 × 0.625 mm	64 × 0.6 mm	320 × 0.5 mm	64 × 0.625 mm	64 × 0.5 mm
	Pitch	0.9844:1	1.0:1	1.0938:1	1.5313:1	0.825:1
Image	Enhanced CT	Yes	Yes	Yes	Yes	Yes
	Thickness	1.3 mm	0.6 mm	0.6 mm	1.3 mm	1.0 mm
	Window	Lung	Lung	Lung	Lung	Lung

The LIDC-IDRI dataset, initiated in 2005 through a collaboration among the National Cancer Institute, seven academic medical centers, and eight imaging companies, is one of the largest publicly available lung CT repositories. Released in 2012, LIDC-IDRI contains 243,958 CT slices (512 × 512) and 7,371 labeled nodules, including 2,669 nodules larger than 3 mm, annotated independently by four expert radiologists. A two-stage annotation process was employed: in the first stage, each radiologist independently recorded the locations of nodules and categorized each lesion into three groups—nodules ≥3 mm, nodules < 3 mm, and non-nodular lesions ≥3 mm—while also scoring the malignancy of each nodule on a 1–5 scale (1 = lowest suspicion, 5 = highly suspicious, 3 = uncertain). In the second stage, all radiologists independently reviewed the annotations from the first stage and made revisions when necessary (15). This study In this study, LIDC-IDRI is used as the source dataset for model training. We included pulmonary nodules with diameters ≥3 mm. Expert consensus masks were generated as segmentation references by parsing the XML annotation files from LIDC-IDRI using the pylidc toolkit (56). Nodules rated 1–2 were defined as benign, those rated 4–5 as malignant, while nodules rated 3 were excluded due to uncertain malignancy. Following common practice, randomly split LIDC-IDRI into training, validation, and internal test sets with a ratio of 8:1:1, corresponding to model optimization, hyperparameter tuning, and internal performance evaluation. The two private datasets serve exclusively as external test cohorts to assess the model's generalization capability and robustness on independent real-world clinical data.

### 4.2 Implementation details

#### 4.2.1 Data preprocessing

All CT volumes were resampled to an isotropic voxel spacing of 1 × 1 × 1 mm^3^. Hounsfield units (HU) were clipped to the range −1,000, 400 to retain relevant pulmonary structures and then normalized to 0, 1 (57). For nodule-focused processing, 3D patches of 64 × 64 × 64 voxels centered on each target lesion were extracted as network inputs. To enhance model robustness with limited training data, we employed data augmentation techniques including random rotation, elastic deformation, random flipping, and Gaussian noise injection. These strategies aim to enrich data variability without altering the fundamental anatomical features of lung nodules, thereby improving the model's versatility and stability across diverse imaging conditions.

#### 4.2.2 Trainning process

All experiments were conducted on one NVIDIA RTX A6000 GPU with 48 GB of memory, using Python 3.10.18 (Python Software Foundation, United States, DE, https://www.python.org/) and PyTorch 2.4.1 (PyTorch Foundation, San Francisco, CA, United States, https://pytorch.org/). During training, all models were optimized using stochastic gradient descent (SGD) with an initial learning rate of 0.01, a momentum of 0.9, and a weight decay of 0.0001. The batch size was fixed at 4 for all experiments, and each model was trained for 200 epochs.

### 4.3 Evaluation metrics

In order to comprehensively evaluate the performance of our model, we employed seven commonly used evaluation metrics, including Dice coefficient (DICE), mean Intersection-over-Union (mIoU), accuracy (ACC), sensitivity (SEN), specificity (SPE), precision (PRE), and F1-score (F1). The calculation methods for these indicators are as follows (Equations 7–13).


$$\begin{array}{l} \mathrm{DICE}\left(A,B\right)=\frac{2\times |A\cap B|}{\left|A|+|B\right|} \end{array}$$ (7)

$$\begin{array}{l} \mathrm{mIoU}=\frac{1}{N}\overset{N}{\underset{k=1}{\sum }}\frac{TP_{k}}{TP_{k}+FP_{k}+FN_{k}} \end{array}$$ (8)

$$\begin{array}{l} \mathrm{ACC}=\frac{TP+TN}{TP+TN+FN+FP} \end{array}$$ (9)

$$\begin{array}{l} \mathrm{SEN}=\frac{TP}{TP+FN} \end{array}$$ (10)

$$\begin{array}{l} \mathrm{SPE}=\frac{TN}{FP+TN} \end{array}$$ (11)

$$\begin{array}{l} \mathrm{PRE}=\frac{TP}{TP+FP} \end{array}$$ (12)

$$\begin{array}{l} F1=\frac{2\text{TP}}{2\text{TP}+\text{FP}+\text{FN}} \end{array}$$ (13)

These metrics were computed based on the numbers of true positives (TP), true negatives (TN), false positives (FP), and false negatives (FN). Detailed descriptions can be found in the referenced literature.

### 4.4 Pulmonary nodule segmentation

To further assess the effectiveness of the proposed method, we compared our multi-task network with several state-of-the-art models designed specifically for either segmentation or classification. For the segmentation task, LNMSNet was evaluated against five representative 3D UNet-based variants, including UNet 3D(18), UNet++ 3D (58), AttentionUNet 3D (59), SwinUNetrV2 3D (60), and ResUNet 3D (61). Table 2 presented the evaluation results. Overall, our model achieved superior performance on the pulmonary nodules segmentation task, with Dice and mIoU scores exceeding those of all competing methods as well as the baseline (82.13 and 69.68%, respectively). Among the comparison models, UNet 3D attained the highest recall (83.34%), and SwinUNetrV2 3D yielded the second highest recall (83.28%). Relative to the baseline ResUNet 3D, our approach improved Dice by 3.14%, recall by 2.07% and mIoU by 2.4%, demonstrating its substantial advantage in delineating pulmonary nodules.

**Table 2:** Comparison of segmentation performance yielded by our LNMSNet to the benchmarking frameworks.

Method	DICE	SEN	PRE	mIoU
UNet 3D (18)	78.75 ± 0.49	**83.34** **±1.42**	78.80 ± 0.39	67.11 ± 0.78
UNet++ 3D (58)	78.98 ± 0.13	82.72 ± 1.74	79.96 ± 1.57	67.18± 0.01
AttentionUNet 3D (59)	78.70 ± 0.30	81.37 ± 2.20	81.04 ± 1.72	66.98 ± 0.57
SwinUNetr-V2 3D (60)	78.78 ± 0.43	83.28 ± 1.84	79.04 ± 1.99	67.02 ± 0.50
ResUNet 3D (61)	78.99 ± 0.04	80.64 ± 0.61	**82.13** **±0.51**	67.28 ± 0.63
LNMSNet (*Ours*)	**82.13** **±0.67**	82.71 ± 0.65	81.55 ± 0.76	**69.68** **±0.98**

The best and second best outcomes are highlighted with bold and underline, respectively.

### 4.5 Malignancy classification

For the classification task, LNMSNet was compared with several widely used 3D classification architectures, including ResNet-18 3D (19), ResNeXt-50 3D (62), PreActResNet-34 3D (63), MobileNetV2 3D (64), and ShuffleNetV2 3D (65). As reported in Table 3, our method outperform other classification methods in almost every metrics. It achieves the highest accuracy of 85.19%, outperforming the baseline 3D ResNet-18 by a margin of 1.97%. Notably, LNMSNet also exhibits superior sensitivity (81.55%), exceeding the second-best method by 3.05%, indicating an improved ability to detect positive cases. Although ResNeXt-50 attains higher precision (87.65%) and specificity (91.14%), our LNMSNet achieved second place in precision (87.11%) and the highest F1-score (84.10%), which demonstrate the overall superiority of our model in the pulmonary nodule classification task. Overall, these results demonstrate that the proposed LNMSNet achieves a more balanced and reliable classification performance compared with competing methods.

**Table 3:** Comparison of classification performance yielded by our LNMSNet to the benchmarking frameworks.

Method	ACC	SEN	PRE	SPE	F1
ResNet-18 3D (19)	83.22 ± 1.98	78.50 ± 4.70	85.12 ± 3.63	87.51 ± 2.86	81.51 ± 2.06
ResNeXt-50 3D (62)	82.44 ± 2.06	72.76 ± 6.34	**87.65** **±4.28**	**91.14** **±2.91**	79.33 ± 3.28
PreActResNet-34 3D (63)	81.44 ± 1.40	74.37 ± 4.99	84.00 ± 4.54	87.69 ± 3.23	78.77 ± 2.97
MobileNetV2 3D (64)	83.62 ± 1.43	77.80 ± 4.18	85.89 ± 6.07	88.80 ± 5.23	81.49 ± 2.57
ShuffleNetV2 3D (65)	81.18 ± 1.00	71.25 ± 1.01	86.02 ± 5.22	90.03 ± 3.23	77.88 ± 1.85
LNMSNet (*Ours*)	**85.19** **±3.14**	**81.55** **±6.57**	87.11 ± 3.82	88.34 ± 5.28	**84.10** **±3.58**

The best and second best outcomes are highlighted with bold and underline, respectively.

### 4.6 Ablation study

To rigorously evaluate the contribution of each core component in LNMSNet, we conducted an ablation study by progressively integrating individual modules into the baseline framework. Quantitative results are summarized in Table 4. It can be observed that the baseline multi-task learning network significantly outperforms the single-task ResUNet 3D in terms of Dice similarity coefficient (80.16 vs. 78.99%). The introduction of the Channel Attention (CA) module increased classification accuracy from 80.35 to 81.61%, while the subsequent addition of the Global Semantic Attention (GSA) module further improved Dice and mIoU by margins of 1.15 and 1.6%, respectively. However, a performance plateau emerged when integrating the Dual Spatial Attention (DSA) module, i.e., classification accuracy peaked at 83.70%, while segmentation Dice accuracy slightly decreased to 80.20%. The complete LNMSNet achieved gains of 1.97% in Dice, 3.82% in Precision, and 2.79% in mIoU. For the classification task, the model achieved an accuracy of 85.19%, sensitivity of 81.55%, specificity of 88.34%, precision of 87.11%, and an F1 score of 84.10%.

**Table 4:** Ablation study for the proposed LNMSNet.

Method	Segmentation				Classification				
	DICE	SEN	PRE	mIoU	ACC	SEN	SPE	PRE	F1
ResUNet 3D	78.99 ± 0.04	80.64 ± 0.61	82.13 ± 0.51	67.28 ± 0.63	–	–	–	–	–
*Baseline*	80.16 ± 0.61	**82.80** **±1.56**	77.73 ± 2.16	66.89 ± 0.85	80.35 ± 0.43	81.53 ± 1.09	78.87 ± 1.05	79.62 ± 5.06	80.06 ± 2.55
+*CA*	80.46 ± 1.78	81.38 ± 2.34	79.83 ± 5.87	67.34 ± 2.51	81.61 ± 1.17	79.39 ± 4.41	83.41 ± 5.72	82.12 ± 3.72	80.60 ± 1.05
+*CA+GSA*	81.61 ± 0.22	80.77 ± 2.95	**82.60** **±2.87**	68.94 ± 0.31	81.49 ± 1.94	71.52 ± 2.02	**90.78** **±2.75**	**87.68** **±4.33**	79.55 ± 2.76
+*CA+GSA+DSA*	80.20 ± 2.33	79.65 ± 3.56	81.01 ± 5.51	66.99 ± 3.20	83.70 ± 5.59	80.96 ± 6.81	85.85 ± 4.40	81.90 ± 2.51	81.38 ± 4.73
*Full* (LNMSNet)	**82.13** **±0.67**	82.71 ± 0.65	81.55 ± 0.76	**69.68** **±0.98**	**85.19** **±3.14**	**81.55** **±6.57**	88.34 ± 5.28	87.11 ± 3.82	**84.10** **±3.58**
+*CA+GSA+DSA+AMS*	82.52 ± 0.25	82.87 ± 0.94	82.19 ± 1.37	70.24 ± 0.35	79.05 ± 1.35	71.39 ± 3.77	84.97 ± 4.02	80.01 ± 3.38	75.41 ± 2.84
+*CA+GSA+DSA+MKDC*	81.11 ± 1.48	79.59 ± 2.17	82.74 ± 3.13	71.29 ± 2.46	82.14 ± 0.85	77.04 ± 2.43	79.67 ± 9.13	80.41 ± 3.29	78.83 ± 2.41

The best and second best outcomes are highlighted with bold and underline, respectively.

To validate the effectiveness and superiority of our multi-scale module against alternatives, we replaced the multi-scale convolutions with AMS and MKDC modules, respectively. Compared to our network, the multi-task network using the AMS module improved DICE by 0.39% with marginal gains in other segmentation metrics. Nevertheless, in the classification component, its accuracy lagged behind our network by 6.14%, with other classification metrics also showing significant disadvantages. The multi-task network using the MKDC module achieved a segmentation DICE score 1.02% lower than ours (81.11 vs. 82.13%) and a classification accuracy 3.05% lower (82.14 vs. 85.19%), while other metrics also fell slightly below our multi-task model.

**Figure 2:**
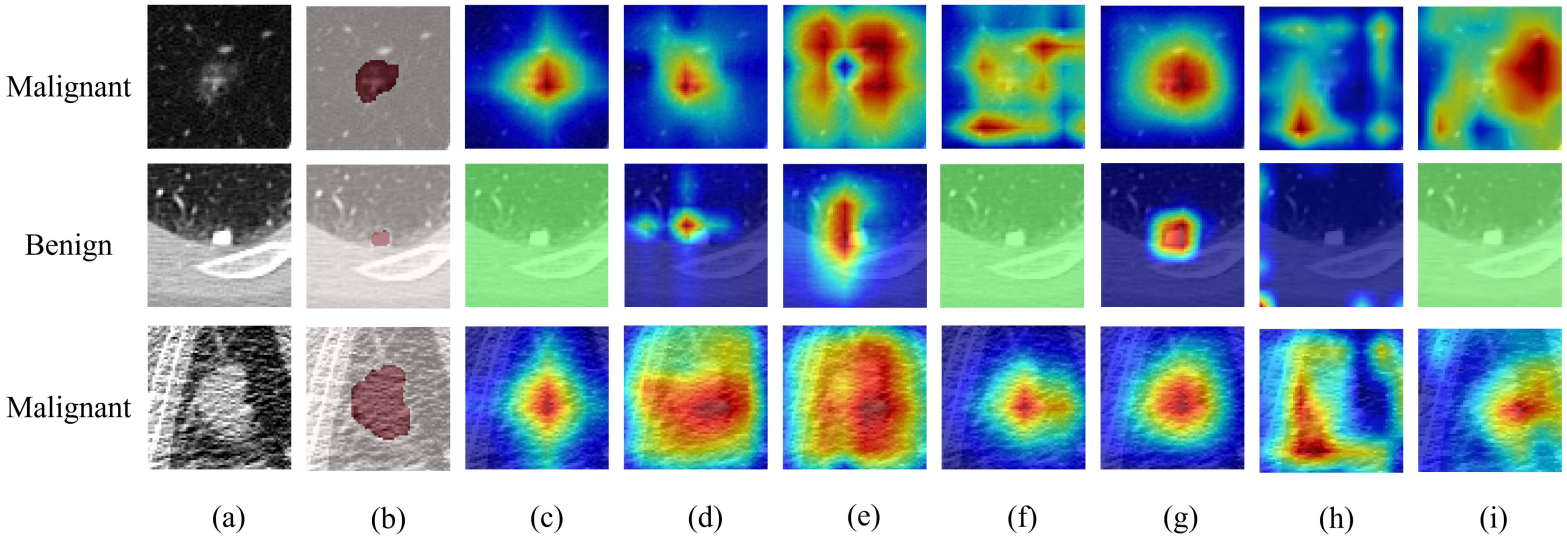
Qualitative comparison of 2D axial attention maps in the ablation study. The visualizations represent the early-fusion features (ffm1) extracted from the third-stage decoder, which are fed back into the classification branch. From left to right, the sub-figures illustrate the progressive enhancement of feature representations: **(a)** original CT slice; **(b)** Ground Truth mask; **(c)** the Baseline model; **(d)** variant with CA; **(e)** variant with CA+GSA; **(f)** variant with CA+GSA+DSA; **(g)** the proposed LNMSNet (Full model with MSConv); **(h)** variant with CA+GSA+DSA+AMS; **(i)** variant with CA+GSA+DSA+MKDC.

Figure 2 displays the heatmap of early fusion features (ffm1) from the decoder. The ablation groups are arranged from left to right: baseline, +CA, +GSA, +DSA, full LNMSNet, and two replacement variants (+AMS and +MKDC). Compared to earlier variants, the full model maintains focus on the lesion core with reduced interference from background responses.

### 4.7 Comparison with multi-task methods

We compared our method with four state-of-the-art multi-task learning networks, including DeepTAAD, CmsVNet, MTAF-Net, and MA-MTLN. The comparative results are summarized in Table 5. In the segmentation task, LNMSNet achieved the best overall performance among all competing methods. Specifically, its DICE coefficient exceeded those of DeepTAAD (50), CmsVNet (53), MTAF-Net (52) and MA-MTLN (51) by 6.83%, 4.73%, 5.08%, and 3.26%, respectively. The remaining Segmentation metrics also consistently ranked first among all compared networks. In the classification task, LNMSNet likewise outperformed the competing methods. Its accuracy (ACC) surpassed DeepTAAD, CmsVNet, MTAF-Net and MA-MTLN by 4.84%, 3.56%, 3.44%, and 2.04%, respectively. Although LNMSNet shows slightly lower specificity (88.34 vs. 88.98%) compared with CmsVNet, it demonstrates clear advantages in sensitivity,precision and F1-score. Taken together, LNMSNet achieves the strongest performance across multiple key segmentation and classification indicators, demonstrating its effectiveness and superiority as an integrated multi-task learning framework.

**Table 5:** Performance comparison with the state-of-the-art multi-task frameworks.

Method	Segmentation				Classification				
	DICE	SEN	PRE	mIoU	ACC	SEN	SPE	PRE	F1
DeepTAAD (50)	75.30 ± 0.79	76.94 ± 4.79	74.18 ± 5.42	60.39 ± 1.01	80.35 ± 3.79	74.89 ± 6.45	85.31 ± 1.75	82.46 ± 3.09	78.47 ± 4.93
CmsVNet (53)	77.40 ± 1.08	75.48 ± 1.49	79.42 ± 1.47	63.13 ± 1.45	81.63 ± 3.06	73.43 ± 7.06	**88.98** **±3.83**	86.36 ± 3.67	79.23 ± 4.35
MTAF-Net (52)	77.05 ± 1.40	76.91 ± 2.37	77.29 ± 3.19	62.69 ± 1.85	81.75 ± 4.15	73.90 ± 7.95	88.89 ± 2.40	86.09 ± 2.77	79.43 ± 5.53
MA-MTLN (51)	78.87 ± 2.20	79.65 ± 5.15	76.40 ± 3.72	63.79 ± 2.93	83.15 ± 4.13	78.02 ± 8.13	87.78 ± 4.53	85.87 ± 3.20	81.60 ± 5.00
LNMSNet (*Ours*)	**82.13** **±0.67**	**82.71** **±0.65**	**81.55** **±0.76**	**69.68** **±0.98**	**85.19** **±3.14**	**81.55** **±6.57**	88.34 ± 5.28	**87.11** **±3.82**	**84.10** **±3.58**

The best and second best outcomes are highlighted with bold and underline, respectively.

### 4.8 Performance evaluation on private datasets

**Figure 3:**
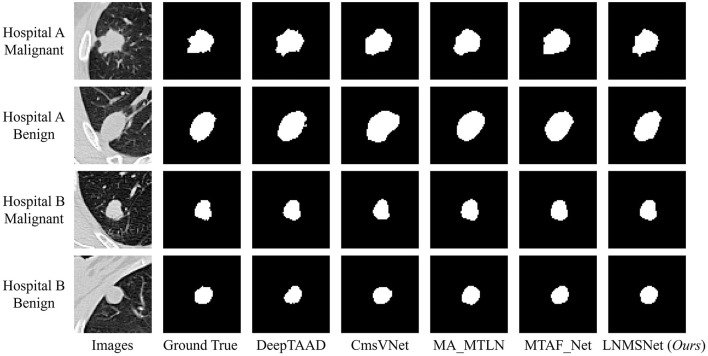
Visualization of segmentation results using different methods on the two private datasets.

To further assess the effectiveness and superiority of our multitask learning framework, we conducted comparative experiments against four advanced multitask networks on two independent private datasets collected from different hospitals. Visual comparison of segmentation results produced by five multi-task models are shown in Figure 3. LNMSNet yields the most accurate lesion boundaries and the closest agreement with the ground truth across all cases. Table 6 The results consistently demonstrate the strong generalization capability of our method across heterogeneous data sources. On the Hospital A dataset, our model achieved a Dice coefficient of 84.31% and an MIoU of 72.87%, surpassing the second-best method, MA-MTLN (81.88%, 69.32%), by 2.43 and 3.55%, respectively. Although our method ranks second in terms of recall and precision, the performance gaps compared to the best-performing model are relatively small, while it maintains clear advantages over the remaining methods. A similar trend was observed on the Hospital B dataset, where our LNMSNet demonstrated even more pronounced advantages. In particular, LNMSNet achieves the best performance among all multi-task methods in terms of Dice coefficient, recall, and mean Intersection over Union (mIoU), with only a slightly lower precision than DeepTAAD. These results indicate that LNMSNet exhibits strong generalization capability and robust segmentation performance across datasets. In the classification task, LNMSNet demonstrated consistently strong performance across both datasets. On the Hospital A dataset, LNMSNet achieved the highest accuracy (65.05%), sensitivity (94.92%), and F1-score (79.15%) among all compared methods, while its precision (67.92%) ranked at an intermediate level among the five approaches. On the Hospital B dataset, LNMSNet achieved the highest accuracy among all compared methods (63.92%), representing an improvement of 5.10% over the best-performing competing model (63.92 vs. 58.82%). In addition, LNMSNet ranked first in sensitivity and F1-score, achieving gains of 5.06% (75.29 vs. 70.23%) and 4.09% (73.29 vs. 69.20%), respectively, while achieving the second-best precision (71.49%). It is worth noting that the specificity values across all multitask methods were generally lower, which can be attributed to the image differences between public and private datasets. Despite this challenge, our method maintained high and stable performance across datasets, further reinforcing its robustness in practical applications.

**Table 6:** Experimental results of the proposed LNMSNet and other benchmarking frameworks on our two private datasets.

Method	Segmentation				Classification				
	DICE	SEN	PRE	mIoU	ACC	SEN	SPE	PRE	F1
Hospital A									
DeepTAAD	81.74 ± 0.72	76.09 ± 2.38	**88.36** **±1.70**	69.12 ± 1.03	65.27 ± 3.67	80.43 ± 4.35	**38.46** **±6.66**	**69.83** **±2.62**	74.72 ± 2.86
CmsVNet	80.07 ± 0.36	77.74 ± 1.50	82.58 ± 1.15	66.67 ± 0.49	62.04 ± 0.80	81.88 ± 3.32	26.92 ± 6.66	66.51 ± 1.26	73.36 ± 0.82
MTAF-Net	80.90 ± 0.60	81.05 ± 2.87	80.89 ± 3.06	67.92 ± 0.84	66.66 ± 1.39	89.85 ± 3.32	25.63 ± 4.44	68.14 ± 0.88	77.48 ± 1.23
MA-MTLN	81.88 ± 0.68	**81.27** **±3.20**	82.64 ± 2.93	69.32 ± 0.97	65.27 ± 1.39	89.85 ± 2.51	21.79 ± 5.88	67.05 ± 1.24	76.77 ± 0.87
LNMSNet (*Ours*)	**84.31** **±0.68**	81.16 ± 1.78	87.73 ± 0.81	**72.87** **±1.01**	**68.05** **±2.41**	**94.92** **±3.32**	20.51 ± 8.01	67.92 ± 1.84	**79.15** **±1.49**
Hospital B									
DeepTAAD	78.45 ± 1.23	70.59 ± 3.24	**88.45** **±2.16**	64.56 ± 1.66	55.29 ± 1.77	52.97 ± 2.25	**59.77** **±4.34**	**71.81** **±2.08**	60.94 ± 1.73
CmsVNet	77.87 ± 0.80	73.38 ± 2.27	83.01 ± 1.51	63.77 ± 1.07	54.31 ± 1.70	60.11 ± 5.08	43.10 ± 5.17	67.11 ± 0.49	63.34 ± 2.86
MTAF-Net	78.60 ± 0.30	75.89 ± 3.48	81.73 ± 3.45	64.74 ± 0.41	56.46 ± 1.56	63.39 ± 3.22	43.10 ± 2.98	68.26 ± 0.85	65.71 ± 1.92
MA-MTLN	79.70 ± 0.67	77.49 ± 3.64	82.24 ± 3.00	66.26 ± 0.93	58.82 ± 2.57	70.23 ± 2.25	36.77 ± 4.34	68.21 ± 1.93	69.20 ± 1.96
LNMSNet (*Ours*)	**82.16** **±0.50**	**78.45** **±2.18**	86.31 ± 1.51	**69.73** **±0.73**	**63.92** **±1.36**	**75.29** **±4.49**	41.95 ± 5.27	71.49 ± 0.75	**73.29** **±1.88**

The best and second best outcomes are highlighted with bold and underline, respectively.

## 5 Discussion

With advances in deep learning-based computer-aided diagnosis, increasing attention has been directed toward improving single-task performance through multi-task learning paradigms. Although numerous methods have achieved promising results in medical image segmentation and classification, accurate delineation and malignancy assessment of pulmonary nodules remain highly challenging due to their small size, blurred boundaries caused by vessel adjacency, substantial heterogeneity, and complex radiological appearance. To the best of our knowledge, there is still a lack of effective studies that jointly refine 3D nodule boundary segmentation and benign-malignant classification using a unified multi-task learning framework. To address this gap, we proposed a 3D multi-task network that integrates segmentation and classification within a shared-encoder architecture, enabling bidirectional feature complementarity. In addition, we established two real-world external datasets to comprehensively evaluate the effectiveness and robustness of the proposed model.

As shown in the previous section, the collaborative learning strategy in LNMSNet achieves accurate segmentation and reliable classification. A key contributor is the proposed MSConv module, which aggregates multi-receptive-field features through parallel convolutional branches and simultaneously captures both local structural details and global contextual cues. Ablation studies further confirm that MSConv is crucial for handling the significant spatial scale variability in lung nodules. While the AMS module offers a rich receptive field and improved feature extraction efficiency, it overlooks edge information due to limited detail capture. The MKDC module, though implementing parallel multi-core deep convolutions, suffers from insufficient context modeling due to its overly broad feature extraction scope. In contrast, the MSConv module strikes a better balance between receptive field size and fine-grained contextual features. While single-scale attention modeling, such as the DSA module, can enhance discriminative features, its limited receptive field makes it susceptible to “focus drift”—where the model tends to over-rely on local noise while overlooking critical lesion regions. By integrating MSConv, feature aggregation is performed on multidimensional morphological features before spatial refinement by DSA. This compensates for potential localization bias in DSA while enriching its feature information, enabling greater focus on lesion areas. Additionally, the GSA and CA modules highlight essential semantic regions and discriminative feature channels, respectively, helping suppress background noise and sharpen lesion boundaries.

Compared with single-task baselines, our multi-task framework yields substantial improvements in both segmentation and classification. This observation suggests that the semantic information from classification assists in boundary localization, while the fine-grained spatial details from segmentation provide richer feature representations for malignancy prediction. However, we also recognize the intrinsic “trade-off” nature of multi-task learning. Sharing an encoder forces the network to balance potentially conflicting objectives; as training proceeds, the model may occasionally prioritize one task over the other. The primary cause is gradient interference. Classification, being a relatively simpler task, tends to converge rapidly with larger gradients, whereas segmentation converges more slowly with weaker gradients. These unequal learning dynamics can result in inconsistent or even opposing gradient directions. Moreover, the two tasks require distinct characteristics—global semantics for classification versus fine-grained spatial details for segmentation—which further exacerbates optimization conflicts. As such, although our method surpasses both single-task networks and state-of-the-art multi-task models in both segmentation and classification performance, how to further mitigate the gradient conflict between tasks remains a primary focus of our future research. Future work will explore dynamic loss balancing and gradient modulation strategies to further optimize our multi-task learning framework. Specifically, we plan to investigate techniques such as GradNorm and dynamic loss weight balancing methods to adaptively harmonize gradient norms across tasks, ensuring equitable weight updates throughout the training process. These advancements will prevent any single task from dominating the optimization, ultimately pushing the performance boundaries of our integrated diagnostic framework even further.

When compared with advanced multi-task learning approaches, our LNMSNet introduces a multi-scale convolution (MSConv) module to ease the conflict between single-scale features in segmentation and classification. By allowing the model to draw on features from multiple receptive fields at once, MSConv improves its ability to handle lesions of different sizes—leading to the best overall segmentation and classification performance. To further validate its clinical relevance, we evaluated LNMSNet on two private datasets comprising nearly 220 cases from two collaborating hospitals. The results demonstrate that our method consistently outperforms competing approaches across both centers. Qualitatively, LNMSNet achieves more precise boundary localization than other methods, particularly in cases with large variations in nodule size, while also exhibiting stronger robustness. Quantitatively, our method maintains its superiority on the private datasets. It is noteworthy that the overall classification accuracy and specificity across all methods remain relatively low. This is likely attributable to the domain shift between the low-dose CT used for training and the contrast-enhanced CT in the private datasets, combined with inherent class imbalance in real-world cohorts. In the future work, we plan to incorporate domain-adaptation strategies to address cross-domain discrepancies and expand multi-center data collection to further enhance model generalizability and reliability.

## 6 Conclusion

In this study, we propose a 3D single-stage multi-task network for joint pulmonary nodule segmentation and malignancy classification. To effectively address the large variability in nodule size and morphology, we introduce the MSConv module, which employs parallel multi-scale convolutional branches to simultaneously capture fine-grained texture patterns and global contextual information, thereby enlarging the receptive field and improving boundary delineation. The model is trained on the LIDC-IDRI dataset and validated on two independent private datasets. Experimental results demonstrate that the proposed LNMSNet consistently outperforms mainstream multi-task learning frameworks on both segmentation and classification tasks and achieves superior segmentation performance compared with state-of-the-art single-task models.

## Data Availability

The raw data supporting the conclusions of this article will be made available by the authors, without undue reservation.
